# Baleen whale cortisol levels reveal a physiological response to 20th century whaling

**DOI:** 10.1038/s41467-018-07044-w

**Published:** 2018-11-02

**Authors:** Stephen J. Trumble, Stephanie A. Norman, Danielle D. Crain, Farzaneh Mansouri, Zach C. Winfield, Richard Sabin, Charles W. Potter, Christine M. Gabriele, Sascha Usenko

**Affiliations:** 1 Department of Biology, Baylor University, Waco, TX 76706 USA; 2 Marine-Med: Marine Research, Epidemiology, and Veterinary Medicine, Bothell, WA 98021 USA; 3 Department of Environmental Science, Baylor University, Waco, TX 76706 USA; 4 Department of Chemistry and Biochemistry, Baylor University, Waco, TX 76706 USA; 5 Division of Vertebrates, Department of Life Sciences, Natural History Museum, London, SW7 5BD UK; 6 Department of Vertebrate Zoology, Smithsonian Institution National Museum of Natural History, Wash, DC 20013 USA; 7 Humpback Whale Monitoring Program, Glacier Bay National Park and Preserve, Gustavus, AK 99826 USA

## Abstract

One of the most important challenges researchers and managers confront in conservation ecology is predicting a population’s response to sub-lethal stressors. Such predictions have been particularly elusive when assessing responses of large marine mammals to past anthropogenic pressures. Recently developed techniques involving baleen whale earplugs combine age estimates with cortisol measurements to assess spatial and temporal stress/stressor relationships. Here we show a relationship between baseline-corrected cortisol levels and corresponding whaling counts of fin, humpback, and blue whales in the Northern Hemisphere spanning the 20th century. We also model the impact of alternative demographic and environmental factors and determine that increased anomalies of sea surface temperature over a 46-year mean (1970–2016) were positively associated with cortisol levels. While industrial whaling can deplete populations by direct harvest, our data underscore a widespread stress response in baleen whales that is peripheral to whaling activities or associated with other anthropogenic change.

## Abstract (web summary)

It has recently been found that stress hormones accumulate in the earwax of whales. Here, the authors use these signatures of stress along with time series of ocean warming and whaling pressure to demonstrate that both stressors were correlated with baleen whale stress over several decades.

## Introduction

For over a century, baleen whales have been subjected to a wide range of anthropogenic disturbances, including: increased shipping, fishing, sound, and numerous pollutants^1–5^. In general, anthropogenic disturbances are known to elicit a stress response in mammals, with increasing evidence of sub-lethal stressors adversely affecting baleen whales^6–8^. Understanding the response to sub-lethal stressors becomes crucial because baleen whales are considered sentinels of their environment and indicators of anthropogenic perturbations^9^. Research involving cetaceans has provided initial evidence that baleen whales respond behaviorally and physiologically to stressors at both a temporal and spatial scale, such as ship traffic or noise^10–14^.

The physiological response to a stressor, defined as the disruption of an organism’s internal environment, or homeostasis, can lead to system-wide health effects from the scale of an individual to the population^6,7^. Despite the inherent challenges associated with measuring the stress response of marine mammals, there is a consensus that cortisol, a stress hormone produced through activation of the hypothalamic–pituitary–adrenal axis, is a proxy of the stress response in mammals and positively correlates with the severity of the stressor^15,16^. Significant challenges hamper direct measurements of cortisol in baleen whales, including sampling cost and logistics, correcting for baseline hormone levels, and the varying longitudinal and temporal resolutions of the tissue or matrix sampled. Techniques have been developed to determine cortisol concentrations from an ever-growing suite of matrices including blood, urine, feces, exhalation, fur/hair and blubber, and some matrices, most notably baleen and cerumen (earwax), provide varying degrees of longitudinal and temporal resolution^17–22^.

Earplugs from baleen whales (Mysticeti) consist of lipid and keratin-based semiannual bands of alternating dark and light laminae (growth layer groups) formed over the life span of the animal^23,24^. While historically used as an aging proxy in baleen whales, earplugs also retain both endogenous (e.g., hormones) and exogenous (e.g., persistent organic contaminants) chemicals within the cerumen, providing the ability to describe lifetime chemical profiles^22,25^. This capacity to archive chemicals along a continuum provides an opportunity to standardize and baseline correct each individual whale’s stress response, revealing a complete retrospective examination of the impacts of potential stressors on free-ranging baleen whales.

Here, we reconstruct lifetime stress profiles from individual baleen whales and assess their relationship to 20th century whaling in the Northern Hemisphere. Specifically, we compare percent change in cortisol from a baseline (baseline-corrected cortisol) with total whaling counts from 1900–1999 to assess whaling as a potential stressor. The term whaling is typically associated with the direct harvest of individual whales, but also encompasses additional anthropogenic extrinsic stressors as well as the ecological consequences of population depletion over time, such as decreased mate abundance. This study represents a link between these sub-lethal, indirect elements of whaling to a physiological stress response in baleen whales.

## Results

### A one hundred and forty six year stress profile

**Fig. 1:**
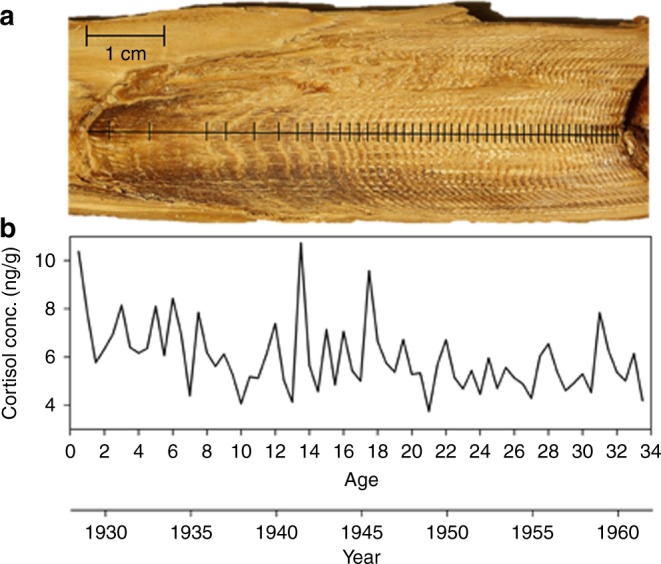
Whale earplug cortisol profile with age and year timeline. **a** Image of a bisected whale earplug with individual laminae identified with black vertical lines. **b** Graph illustrates reconstructed lifetime cortisol profile (ng/g) as a function of age and year

**Fig. 2:**
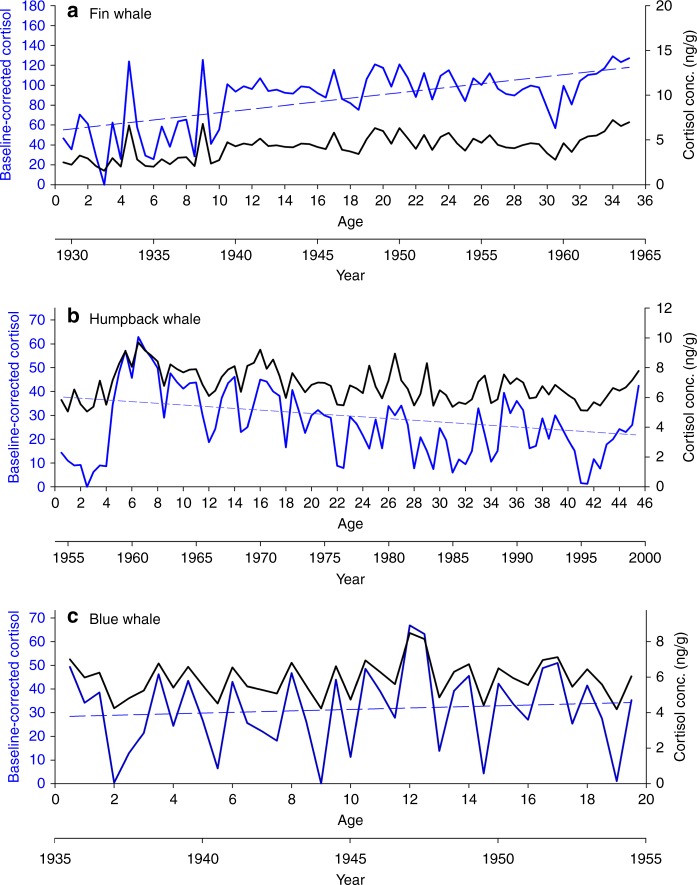
Baseline-corrected cortisol as a function of age and year. Reconstructed lifetime cortisol concentrations (ng/g; black line) and baseline-corrected cortisol (blue line) profiles for a **a** 35-year-old fin whale with a positive lifetime stress trend (dashed blue line), **b** 45-year-old humpback whale with a negative lifetime stress trend (dashed blue line), and **c** 19-year-old blue whale with no lifetime stress trend (dashed blue line)

We report 146 years (c. 1870–2016; *n* = 1084 laminae) of cortisol (ng/g), standardized to baseline-corrected cortisol, to produce lifetime stress profiles from 20 mysticete earplugs representing three species: fin (*Balaenoptera physalus*), humpback (*Megaptera novaeangliae*), and blue (*Balaenoptera musculus*) whales (Fig. 1; *n* = 12, 4, and 4, respectively) from the Northern Hemisphere. Eight earplugs sampled from whales originating in the Pacific Ocean had an estimated overall mean age of 31.4 ± 24.2 years ± standard error (± SE) (*n* = 5 males, mean age 16.8 ± 16.5 years; *n* = 3 females, mean age 55.8 ± 9.2 years), whereas twelve earplugs from Atlantic Ocean stocks had an overall estimated mean age of 24.9 ± 14.1 years (*n* = 6 males, mean age 19.9 ± 10.3 years; *n* = 6 females, mean age 28.9 ± 17.0 years). Cortisol concentrations (ng/g) were determined for each extracted lamina (growth layer representing 6 months), and lifetime profiles were subsequently baseline-corrected for comparison between individuals and among species (Fig. 2). When comparing cortisol levels among species and between sexes, mean baseline-corrected cortisol was significantly highest in fin whales (fin > humpback > blue) (pairwise comparison with Tukey’s method to account for multiple comparisons; *t* = 5.83; *P* value < 0.001), whereas by sex, male humpback whales had the highest cortisol when compared to blue and fin whales (humpback > blue > fin; mixed-effects linear regression followed by pairwise comparison with Tukey’s method to account for multiple comparisons; *t* = 6.49; *P* value < 0.001; Table 1). There was, however, a significant interaction between sex and age when assessed together in the final regression model (*P* value < 0.05).

**Table 1:** Baseline-corrected cortisol, mean/median, and standard deviation (± SD) for large whale earplugs (*N* = 20)

Whale species and sex (*n*)	Percent from baseline mean/median (SD)
Fin whale	
All (12)	63.48/57.34 (39.58)
Male (8)	52.78/43.72 (39.86)
Female (4)	77.66/78.21 (34.5)
Humpback whale	
All (4)	43.73/38/35 (31.17)
Male (1)	96.30/84/76 (49.44)
Female (3)	37.57/34.42 (21.14)
Blue whale	
All (4)	48.53/37.00 (37.71)
Male (2)	77.02/86.45 (39.14)
Female (2)	30.66/25.18 (23.11)

### Whaling numbers and cortisol

**Fig. 3:**
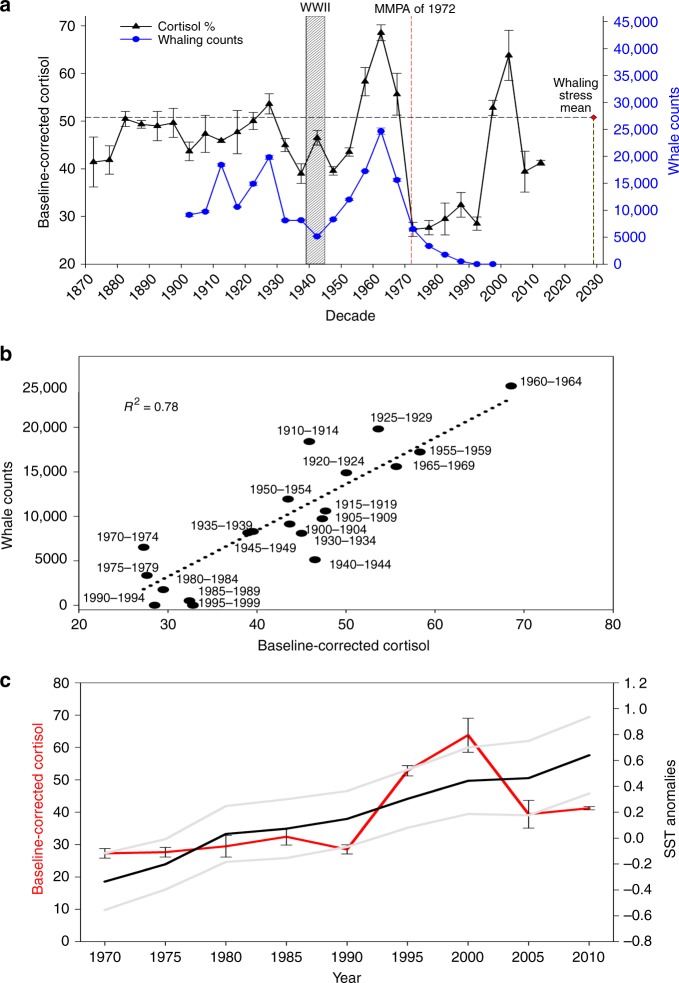
Whale cortisol relationship with whaling numbers and sea-surface temperature. **a** Mean baseline-corrected cortisol (± SE) with corresponding whaling counts (± SE) of the 20th century for blue, fin, and humpback whales in the Northern Hemisphere. Striped bar corresponds to World War II years (1939–1945). For reference, the red dashed line connotes the Marine Mammal Protection Act of 1972. *N* = 1084 lamina; 1870s, *n* = 17; 1880s, *n* = 20; 1890s, *n* = 20; 1900s, *n* = 35; 1910s, *n* = 32; 1920s, *n* = 71; 1930s, *n* = 144; 1940s, *n* = 225; 1950s, *n* = 212; 1960s, *n* = 89; 1970s, *n* = 60; 1980s, *n* = 60; 1990s, *n* = 60; 2000s, *n* = 27; 2010s, *n* = 12). Whaling counts (total deaths) and ±SE calculated from Rocha et al.^26^. **b** Relationship between 5-year mean baseline-corrected cortisol (± SE) and 5-year mean whaling counts (± SE) for the 20th century (*r*^2^ value = 0.78). **c** 1970–2016 baseline-corrected cortisol (± SE; red line) and SST anomalies (1971–2000^35^; black line with 95% confidence interval as gray; *r*^2^ value = 0.46). SE standard error of the mean

The association between total whaling counts during the 20th century^26^ and baseline-corrected cortisol concentrations (Fig. 3a) was evaluated collectively for all three whale species (years 1900–1999 *n* = 942 laminae; Fig. 3b; *r*^2^ value = 0.78; Table 2). Commercial whaling increased in scope and became increasingly more efficient during the 20th century, resulting in a 45% increase in fin whale harvests and a 70% increase in humpback harvests when compared to the 19th century^26^. This efficacy was maintained during the decade of the 1930s, where ∼50,000 fin, humpback, and blue whales were harvested from Northern Hemisphere waters^26^. During this period, between world wars, maximum baseline-corrected cortisol levels were achieved (53% above baseline) in earplugs, mirroring maximum harvest take of whales (Fig. 3c). Interestingly, the years 1939–1945 (World War II; WWII) revealed a departure from the close association between mean cortisol concentrations and mean whaling harvests (*n* = 10 earplugs, *n* = 225 laminae; Fig. 3a, b). Specifically, during the WWII era, baseline-corrected cortisol within earplug laminae increased 10% while whaling harvests decreased to the lowest numbers observed during the pre-whaling moratorium era (pre-1986) in the Northern Hemisphere (Fig. 3a). While it remains unknown if a 10% increase in baseline-corrected cortisol represents an adverse physiological or behavioral response, the departure from the close association with whaling counts may be in response to other stressors such as marine-based wartime activities. In other words, the stressors associated with activities specific to WWII may supplant the stressors associated with industrial whaling for baleen whales. Therefore, we surmise that wartime activities (e.g., underwater detonation of ordinance, naval battles including ships, planes, and submarines), as well as increased vessel numbers, contributed to increased baseline-corrected cortisol concentrations during this period of reduced whaling. With the extensive migration patterns of baleen whales, interaction with widespread wartime activities would seem plausible and deleterious to baleen whales^27–29^.

**Table 2:** Results of fitting a linear mixed-effects model to determine the level of baseline-corrected cortisol in earplug of large baleen whales (1900–1999) given sex, age, sex/age interaction, and number harvested over 5-year intervals

Variable	Coefficient	SE	Wald *z*	*P*-value
Percent cortisol above baseline				
Sex (female versus male)	−0.461	12.700	−0.04	0.971
Age category (years)				
11–24.9	9.789	3.288	2.98	0.003
≥ 25	19.054	5.949	3.20	0.001
Sex/age category interaction	−9.880	3.4424	−2.89	0.004
Number of whales harvested each year	0.005	0.002	3.18	0.001

SE, standard error

Age = <11, 11–24.9, ≥ 25 years. The reference values were sex = male, age category = <11 years. Significant predictors are those with *P* values ≤ 0.05

Once post-war whaling resumed in the 1950s, the number of whales harvested drastically increased and peaked in the early 1960s, with nearly 150,000 animals harvested^26^. During the early 1960s, baseline-corrected cortisol peaked to a maximum 68%, denoting the highest mean baseline-corrected stress values over the 20th century (Fig. 3a). Comparing 5-year mean groupings in baseline-corrected cortisol, the lowest 5-year means spanned the years 1970–1999, whereas the highest 5-year means occurred during the 1920s, 1950s, and 1960s (Fig. 3b).

A decline in both whaling numbers (7.5% yr^−1^) and a corresponding decrease in cortisol levels (6.4% yr^−1^) occurred during the mid-1970s, when protective whaling moratoriums were adopted in the United States (Marine Mammal Protection Act of 1972 = red line, Fig. 3a). Whaling harvest counts decreased 75% for fin, humpback, and blue whales in the Northern Hemisphere during this period^26^. This period of dwindling harvest counts coincided with the lowest baseline-corrected cortisol concentrations measured from whale earplugs over the 20th century (27% above the baseline value; Fig. 3a, b). We propose that this close association between baseline-corrected cortisol concentrations and whaling harvests indicates a relatively prompt response by baleen whales to the direct and indirect activities of whaling. From the 1970s through the 2010s (*n* = 132 laminae; *n* = 6 earplugs), as whaling counts were reportedly zero in the Northern Hemisphere, mean baseline-corrected cortisol levels steadily increased (Fig. 3b), with recent peaks reaching near the maximum levels observed before the whaling moratorium (Fig. 3b).

### Recent anthropogenic stressors

Recent modeling efforts estimate the impact of several anthropogenic drivers toward ecological change with sea-surface temperature (SST) anomalies (1985–2005) as the most pronounced stressor in marine ecosystems^1,30^. According to the Intergovernmental Panel on Climate Change, the ocean has warmed on a global scale, by an average of 0.11 °C per decade from 1971 to 2010^31^. To assess the increases in stress in baleen whales and to evaluate specific anthropogenic stressors, an anthropogenic pressure index specifically for baleen whales (API_w_) was generated (Supplementary Table 1, Supplementary Table 2). It is important to note that there are significant gaps in longitudinal datasets for several anthropogenic stressors assumed relevant for baleen whales, including recreational fishing, tourism, non-cargo shipping, sea ice extent, freshwater input, disease, and point-source pollution. However, data of SST anomalies span similar time periods for samples used in this study. For whales, SST is an important ecological factor, affecting prey aggregates, thermal constraints, and migratory markers^11,13,32^. Specifically for cetaceans, SST anomalies have been linked to a number of factors, including changes in habitat preferences, competitive interactions between species, and changing geographic ranges^33^. Baseline-corrected cortisol levels positively associated with SST anomalies from 1970 to 2016 (*r*^2^ value = 0.46; simple linear regression, Fig. 3c), indicating that the increased frequency in SST anomalies replaced whaling as a stressor.

### Modeling

This 146-year cortisol dataset represents the longest temporal record of stress in baleen whales, and therefore presents an opportunity to model the potential influence of a wide range of abiotic, biogenic, and anthropogenic factors. For example, in linear regression (Wald chi-squared test statistic) analyses the predictor variables of age (6.40; *P* value = 0.041), yearly whale harvest counts (25.95; *P* value < 0.0001), and interaction term between sex and age (2.96; *P* value = 0.222) were each independently associated with baseline-corrected cortisol at the preset cutoff of *P* value < 0.25, and were thus included during model building and evaluation (Supplementary Table 3). Sex by itself was not significantly associated with cortisol (0.370; *P* value = 0.542), but was included in the multivariate model since it was part of the interaction term. Though the SST anomalies variable alone was not significantly associated with cortisol (0.120; *P* value = 0.734), it was still included in the final model building steps as a potential predictor of cortisol levels. The remainder of the covariates tested did not show evidence of being associated with baseline-corrected cortisol. Likelihood ratio tests indicated that sex was not a significant predictor of baseline-corrected cortisol in the presence of the other covariates; however, it was shown to significantly interact with age and was thus included as part of an interaction term in the final adjusted model (Supplementary Table 3). Baseline-corrected cortisol significantly differed between the sexes as age progressed (Supplementary Figure 1). The final model selected, which included sex, age, sex/age interaction, and number of whales harvested each year, was determined to be the most parsimonious model (Model B—Supplementary Table 3; Table 2). Although the variables sex and age did not independently explain the observed deviations in baseline-corrected cortisol (Model E—Supplementary Table 3), their importance in the final model indicates these two variables do account for some of the variation in cortisol levels not accounted for by whaling harvest counts. The significant negative coefficient for the interaction variable (sex/age) indicates that, overall, females in this study experienced less stress than did males over their lifetime. Specifically, mean baseline-corrected cortisol was ∼12% less for females over a lifetime. In other words, adult males had significantly elevated baseline-corrected cortisol levels (*P* value = 0.004; linear mixed-effects model, Table 2; Supplementary Figure 1).

In univariate regression analysis, the relationship between yearly whale harvest counts and baseline-corrected cortisol was independently significant (*P* value = 0.001). After including yearly whale counts in the final multivariate model, this positive relationship continued to be significant, with baseline-corrected cortisol increasing as the number of harvested whales increased (Fig. 3b, Table 2). For example, when yearly harvested whales numbered in the mid-range of 7000–9999 (corresponding to the decades: 1900s, 1930s–1940s), the baseline-corrected cortisol was higher compared to yearly harvest counts numbering < 7000 (corresponding to the mid-1970s–2010s) and less than those yearly totals corresponding to higher harvest levels (>10,000 during the 1910s, 1950s, and 1960s).

We show a positive relationship between activities associated with commercial whaling harvests and baseline-corrected cortisol from baleen whale earplugs spanning the 20th century (Fig. 3b). While industrial whaling is known to deplete populations by direct harvest, our data underscores that stress was not only a result of direct harvest on targeted individual whales, but also increased in whales not directly harvested. Although unable to account for the individual components of whaling effort through time such as changes in whaling methodologies and number of ships, our results illustrate a critical link between whaling and stress in baleen whales. Combining multiple life history profiles characterized the effect of whaling on baseline-corrected cortisol in baleen whale populations; however, it should be noted that datasets from individual whales exhibit the variability of a complex life history (Table 3, Fig. 2). Taken individually, these life history profiles cannot easily capture temporal patterns or anthropogenic events.

**Table 3:** Mean cortisol as a percent change from baseline (baseline-corrected) and standard error (± SE) for fin, humpback, and blue whales in the N. Hemisphere as related to location (ocean basin), sex, estimated age (years), and life span

Species	Location	Sex	Age estimates (years)	Lifespan	Mean %BL cortisol ± SE	%BL cortisol range	%BL cortisol *r*^2^ slope
Fin	Pacific	M	35	1929.5–1964	99.9 ± 7.5	2.6–187.7	0.67
	Pacific	M	33.5	1928.5–1961.5	43.3 ± 5.6	9.7–198.7	0.20
	Atlantic	M	22.5	1933–1955	39.9 ± 2.4	1.2–67.9	0.13
	Atlantic	M	38.5	1871.5–1909.5	48.2 ± 2.5	7.8–103.7	0.08
	Atlantic	F	13	1943.5–1956	29.1 ± 3.6	7.4–78.2	0.02
	Atlantic	F	19	1936.5–1955	68.8 ± 4.3	1.6–116.4	0.00
	Atlantic	F	53	1902.5–1955	78.7 ± 2.5	6.0–129.1	0.02
	Atlantic	F	48	1915.5–1963	69.4 ± 3.2	11.5–126	0.20
	Atlantic	M	11.5	1918.5–1929.5	38.4 ± 6.8	8.9–143.9	0.12
	Pacific^a^	M	2	2013–2015	3.3 ± 2.3	1.4–10.1	0.40
	Pacific^a^	M	1.5	2013.5–2015	8.2 ± 7.9	2.6–24.0	0.95
	Atlantic	M	18.5	1948.5–1964.5	25.2 ± 2.0	2.4–60.1	0.08
Humpback	Pacific^a^	F	45.5	1954.5–1999.5	26.7 ± 1.7	1.3–47.9	0.16
	Pacific^a^	F	59	1955–2014	28.6 ± 1.6	1.9–58.2	0.08
	Pacific^a^	F	63	1940–2003	41.8 ± 1.2	14.5–69.2	0.16
	Atlantic	M	16.5	1948.5–1964.5	89.5 ± 10.2	2.5–187.9	0.08
Blue	Atlantic	F	19.5	1935.5–1954.5	24.8 ± 2.7	1.4–60.3	0.00
	Atlantic	F	21	1934.5–1955	42.6 ± 4.8	3.3–110.8	0.19
	Atlantic	M	11.5	1937–1948	55.8 ± 8.0	8.7–111.5	0.01
	Pacific^a^	M	12	1995–2007	63.9 ± 7.1	43.9–156	0.13

%BL cortisol *r*^2^ slope denotes cortisol linear regression value over lifetime of whale. ^a^denotes recent earplugs

Analytes extracted from an earplug lamina represent a 6-month mean; therefore, the relatively close association between stress and whaling counts during the 20th century would suggest a seemingly close relationship and rapid physiological response to direct or indirect threats. As detailed by Boonstra^6^, the mammalian stress response and homeostatic set-point are not fixed, but modified by experience. Experiences may alter or modulate the stress-axis response, and under conditions where the stressor becomes chronic (e.g., days to months), the normal suppressive effects of glucocorticoids weaken. Interestingly, the overall trends in baseline-corrected cortisol from earplugs in this study did not moderate with time (Fig. 3a), possibly indicating a conserving of the stress response. In other words, these whales closely mirrored the stress of their surroundings. This reinforces the concept of large baleen whales as sentinels of the marine environment^9^. Additionally, the intercept of the association between the 5-year mean whaling counts and baseline-corrected cortisol is ∼20%, which may represent the basal level of cortisol in the absence of whaling.

The influence of increased SST anomalies on baseline-corrected cortisol in the present study is insignificant prior to the 1970s (*r*^2^ value = 0.01). However, from the 1970s to 2016, increased SST anomalies were positively associated with baseline-corrected cortisol (Fig. 3c; *r*^2^ value = 0.46). Currently, changes in SST anomalies appear to be one of the dominant drivers of cumulative anthropogenic impacts on marine ecosystems^30^, though other anthropogenic stressors, such as increased sound, pollutants, over-fishing, and ocean acidification likely contribute to increased cortisol in baleen whales. As more earplugs from current whale mortalities are collected, the association between anthropogenic drivers and their effects on large baleen whales can be refined.

One of the most important challenges researchers and managers confront in conservation-based ecology is predicting how populations will respond to sub-lethal natural, environmental, and anthropogenic stressors, as well as the cumulative effect of these stressors^8^. This study illustrates the importance of using matrices such as earplugs to reconstruct lifetime profiles, determine baseline hormone values, and model retrospective data to determine potential associations with past or present anthropogenic stressors. While this study focused on the stress hormone cortisol in baleen whales and its relationship to potential anthropogenic stressors through time, measuring additional hormones or analytes offers an opportunity to examine the impacts of chronic stress on whale behavior including migration patterns through stable isotope analysis and fecundity through progesterone measurements.

Long-lived organisms endure evolutionary pressures resulting from a rapidly changing environment to which whales must quickly adapt^34^. Given the current pace of change over the past century, this capacity, or plasticity, to respond promptly may be compromised. Current anthropogenic stressors may have a greater deleterious effect to species already compromised by the significant population declines associated with a 100 + years of industrial whaling. While we established an association between industrial whaling and stress in baleen whales over the 20th century, additional anthropogenic factors, such as recent SST anomalies due to climate change, increased fishing and krill harvests, and sea ice decline should be considered and further studied during 21st century post-whaling in the Northern Hemisphere.

## Methods

### Earplug acquisition

A total of 1084 earplug lamina from three species, spanning 146 years, yielded a subset of laminae (*n* = 942) corresponding with published 20th century whaling counts (1900–1999)^26^. Baleen whale earplug samples were originally collected during whaling activities during the 20th century (pre-1972) and subsequently archived in museums (Smithsonian Museum of Natural History or Natural History Museum of London) or extracted from recent stranded carcasses (Table 3).

### Aging

Baleen whale earplugs were weighed and measured before storing in a −30 °C until processing. After removal from the freezer, all whole earplugs were bisected using medical grade zirconia ceramic scalpel blades or ceramic knives. A frozen bisected earplug was polished using progressively finer grain sandpaper (80–600 grit), rinsed using deionized water and photographed using a Canon DSLR camera with macro lens (Canon 6D Mark II). The SLR camera was mounted to a copy stand with adjustable fluorescent lighting (Bencher Copymate). Digital photographs were taken and used to age the whale by counting the number of dark and light laminae, assuming the combination of one dark and one light lamina (growth layers) constitutes one year of life^23^. Before delamination, two independent readers used digital photographs to age each earplug included in this study. One additional reader was used if there was greater than a ± 5% discrepancy in the total laminae counts.

### Sectioning whale earplugs

Baleen whale earplugs (*n* = 20; Table 3) were meticulously delaminated (*n* = 1084) using a ceramic scalpel under magnification (head-mounted magnifier loupe (10–20×)). Each separated lamina was weighed to the nearest 0.001 g, placed into a glass vial, filled with nitrogen and stored at −30 °C until processed.

### Stress-related hormone radioimmunoassay technique

Cortisol concentrations were determined in each lamina using an enzyme-linked immunosorbent assay (ELISA; Enzo Life Sciences; ADI-900-071). During the extraction phase, a minimum of 15 mg of each lamina was added to 2 ml of diethyl ether, vortexed for 30 s and subsequently centrifuged for 30 s. A series of intralaboratory experiments determined 15 mg was the minimum amount of lamina material needed to produce consistent results. The resulting supernatant was pipetted into a separate vial and stored. This process was repeated twice in which time the supernatant was placed under a nitrogen stream until dried, then capped and stored in a 4 °C refrigerator. The waxy pellet was suspended using 250 μl of assay buffer into a vial and vortexed for 30 s. Cortisol extractions were assayed using an ELISA kit (ENZO catalog # 80-0010) and validated for linearity and accuracy^18^. Each extraction was run in duplicate on a 96-well microtiter plate (Beckman Coulter DTX 880 Multimode Detector). The cortisol in the sample binds competitively with the enzyme conjugated to cortisol. An added substrate detects enzyme activity resulting in an inverse relationship between optical density and the amount of cortisol in the sample. This inverse relationship was calculated using a standard curve with results converted to ng/g. Intra-assay precision was determined by taking samples containing 156, 1250, and 5000 pg/ml concentrations of cortisol and running these samples multiple times within each assay. Sample recoveries for cortisol were measured using duplicate cerumen aliquots.

### Technical approach

We assessed the relationship of whaling and stress by evaluating 5-year mean whale counts with corresponding mean baseline-corrected cortisol (simple linear regression). To identify environmental and anthropogenic factors that were independently associated with baseline-corrected cortisol in the cerumen, multivariate longitudinal random (mixed-effects) linear regression models were used to assess the relationship between cortisol levels and nine independent predictor variables. The variables included species, sex, age, sex/age interaction, ocean basin (Pacific or Atlantic), proportion of each species harvested by ocean basin, the geometric mean decadal proportion harvested for each species (1900–1999), the number of whales harvested each year (1900–1999), and SST anomalies (deviations from mean SST 1971–2000) (Supplementary Table 4). Because historical data for number of whales harvested were not available prior to 1900 or after 2000^26^, only laminae for the years 1900–1999 were included in the regression modeling. Beyond whaling (total whaling counts), we attempted to account for the effect of additional anthropogenic stressors on cortisol levels such as pollution, fishing pressure, natural resource exploration, and noise. However, with the exception of SST anomalies, these additional anthropogenic variables were not incorporated into the present model due to insufficient longitudinal data^1,30^. Nevertheless, the value of incorporating other potential anthropogenic factors into future modeling efforts should include appropriate anthropogenic stressor indices to help explain increased cortisol. The steps involved in developing a potential API_w_ for future analyses are described in the Supplementary Table 2 using currently available anthropogenic stressor data (2008–2013) and whale earplugs from this study^1,30^.

The remaining, unexplained cortisol variation was partitioned into that attributable to systematic differences among individual whales (interwhale variation) and that attributable to variations along the trajectory of measurements for an individual (intrawhale variation). A random effect, used to account for variation across individuals, was included as a variable in the models. By including random intercepts in the regression models, this approach also accounted for correlations between repeated cortisol measurements for each whale. The random intercepts were whale-specific terms indicating that each whale had a different starting value of cortisol and that the correlation matrix varied from one whale to another. Therefore, the parameter estimates involved whale-specific interpretation; that is, as an example, coefficients can be interpreted as a change in percent baseline-corrected cortisol per unit change in the covariate. Additionally, models were used to check for the presence of interaction between explanatory variables that might result in the simultaneous additive influence of two variables on a third such as interaction between sex and age.

Whaling effort was not temporally or spatially uniform over time due to changes in methodology, species abundance, and other factors^26^. Because it was not possible to directly quantify whaling effort such as number of ships, land-based stations, or effort at sea, harvested whale counts served as a proxy for whaling effort in the models. Independent main effect predictor variables for whaling effort were incorporated into the mixed-effects models using harvest counts obtained from previously published catch data using three methods^26^: (1) proportion by ocean in Northern Hemisphere—proportion of each species harvested out of all species total in each of the two Northern Hemisphere oceans (Pacific and Atlantic); (2) geometric mean proportion harvested over decades (1900–1999)—mean of each species-specific decadal proportion harvested out of all whale species harvested in the Northern Hemisphere. Specifically, for each decade, each species’ catch total was divided by the total of all Northern Hemisphere species. That proportion was multiplied by the total number of catches in the Northern Hemisphere for all species (*n* = 830,089) resulting in number caught for each species for each decade. The numbers caught for each species were added over the decade and divided by the Northern Hemisphere catch total, resulting in a proportion for each species over the ten decade time period. Northern Hemisphere decadal whaling data for this proportion were not listed by ocean basin (Pacific or Atlantic) in the original source^26^, so could not be further detailed; (3) yearly total number of whales harvested for all hunted species in the Northern Hemisphere covering the study period (obtained from Table 4 in Rocha et al.^26^). Collinearity between these three whaling effort variables was assessed to aid in selection of one to be included in the final model. Since the whales in the present study all originated from the Northern Hemisphere, direct comparisons to Southern Hemisphere whaling counts were not evaluated.

Recent studies have identified deviations in SST anomalies as an important driver in cumulative anthropogenic stress, therefore, these data were added as a covariate in the analyses^1,30^. Deviations data were obtained from the United States National Oceanic and Atmospheric Administration (NOAA) and were categorized into seven deviation levels ranging from −0.90 to 0.50 with approximate corresponding years for each category^35^.

Univariate models were developed to assess the individual relationship between each predictor variable and the response variable (baseline-corrected cortisol). Those predictor variables associated with baseline-corrected cortisol at a P value of ≤ 0.25 in univariate analysis were included in the initial full model, as well as those variables of biological interest or identified in the literature as potential covariates. Each of the covariates was individually removed from the full and reduced models as needed to form the most parsimonious model based on likelihood ratio tests and Akaike Information Criterion (Akaike 1973)^3,6^. If the removal of a variable caused another variable’s coefficients to change more than 10%, then the latter variable was retained, indicating the model was sensitive to its inclusion. Estimation of partitioning factors was by restricted maximum likelihood using the Stata “xtmixed” command for mixed-effects models (Stata Corp., College Station, Texas 77845, USA).

Mean baseline hormone concentration was determined by averaging the lowest three cortisol concentrations within each individual earplug. Using this average value, percent change from mean baseline value (absolute value, baseline-corrected) was calculated for each subsequent time point within each earplug over the entire whale life span by the formula

1 $$\% {\mathrm{BL}} = \left( {C_i-C_{{\mathrm{BL}}}} \right)/\left( {\left( {C_i + C_{{\mathrm{BL}}}} \right)/2} \right)\times100$$

with *C*_i_ cortisol concentration (ng/g) and *C*_BL _= the mean baseline value for the individual earplug. To evaluate potential uncertainty in baseline values, a sensitivity analysis was conducted by calculating mean values for additional concentration pools using the lowest three and lowest five cortisol concentrations from five individual whales from three species. The sensitivity analysis revealed that the lifetime regression trend was identical among the three groupings (lowest, mean lowest 3, and mean lowest 5), however, using the mean lowest three concentrations as a mean baseline value resulted in lowest mean error from percent change from baseline. Modeling (see above) was used to assess the relationships of species, age and time (based on age/lamina counts and collection dates) with baseline-corrected cortisol among all earplugs sampled. 20th century whaling data was obtained from Rocha et al.^26^ and used to fit with mean 5-year cortisol baseline data over the same period (least linear squares). The standard error of the mean (±SE) was calculated where *σ* is the standard deviation (±SD) of the population and *n* is the number of earplugs.

## Electronic supplementary material

Supplementary Information

## Data Availability

All relevant data are available from the authors upon reasonable request.
