# Supplementary material for: Baleen whale cortisol levels reveal a physiological response to 20th century whaling
Source: Nat Commun. 2018 Nov 2;9:4587. doi: 10.1038/s41467-018-07044-w (PMC6215000; doi:10.1038/s41467-018-07044-w)
Supplement: Supplementary file 1 — Supplementary Information [file 41467_2018_7044_MOESM1_ESM.pdf]

Baleen whale cortisol levels reveal a physiological response to 20th century whaling

Trumble et al.

Supplementary Data for Baleen whale cortisol levels reveal a physiological response to 20th century whaling

Authors: Stephen J. Trumble[1*], (254)710-2128, Stephen_Trumble@baylor.edu

Stephanie A. Norman[2], (206)321-0249, stephanie@marine-med.com

Danielle Crain[1], (254)710-2559, Dani_Crain@baylor.edu

Farzaneh Mansouri[3], (254)710-2303, Farzaneh_Mansouri@baylor.edu

Zachary Winfield[4], (903)238-3075, Zach_Winfield@baylor.edu

Richard Sabin[5], +44(0)20-7942-5206, r.sabin@nhm.ac.uk

Charles Potter[6], (202)633-1261, POTTERC@si.edu

Christine Gabriele[7], (907)697-2664, chris_gabriele@nps.gov

Sascha Usenko[1,3,4,*], (254)710-2302, Sascha_Usenko@baylor.edu

Affiliations:

[1]Department of Biology, Baylor University, Waco TX, 76706, USA

[2]Marine-Med; Marine Research, Epidemiology, and Veterinary Medicine, Bothell WA 98021 USA

[3]Department of Environmental Science, Baylor University, Waco TX 76706, USA

[4]Department of Chemistry and Biochemistry, Baylor University, Waco TX 76706, USA

[5]Division of Vertebrates, Department of Life Sciences, Natural History Museum, London, SW7 5BD, U.K.

[6]Department of Vertebrate Zoology, Smithsonian Institution National Museum of Natural History, Wash DC 20013 USA

[7]Humpback Whale Monitoring Program, Glacier Bay National Park and Preserve, Gustavus, AK 99826, USA

Due to insufficient global data on several key drivers known to significantly impact marine ecosystems (i.e., recreational fishing, disease, coastal habitat alteration, non-cargo shipping, changes in sedimentation, changes in sea ice extent, freshwater input, tourism, and point-source pollution), they were not included in the indices[1,2]. Anthropogenic drivers were weighted by their estimated ecological impact. Due to the wide-ranging distribution and migratory patterns of most baleen whales, differences in global cumulative impact scores between 2013 and 2008, of large marine ecosystems (LMEs) encompassing all potential habitat of each whale species, were used[1,2]. For example, most stocks of humpback whales are highly migratory, traveling to high latitude feeding grounds in the summer, then returning to calving grounds in subtropical or tropical waters[3]. Thus the differences in scores between 2013 and 2008 for this species were derived by averaging the mean cumulative impact scores across all LMEs most commonly utilized by the whale, resulting in a mean change in cumulative impact score over time for 12 anthropogenic stressors (Supplementary Table 1, Supplementary Table 2)[1,2]. The LMEs overlapping each whale's habitat were based on currently known distribution data for breeding and feeding grounds (Supplementary Table 2). If whaling (or stranding) location of the animal were known, LMEs were selected based on the most likely feeding and breeding habitat(s). In cases where specific location of whaling take or stranding were unknown beyond ocean (Pacific or Atlantic), then impact scores from all possible LMEs potentially used by the whale were included in the average. The 12 stressors were based on those previously calculated and mapped for global marine ecosystems[1,2]. Since the median for the average differences in cumulative impact scores between 2013 and 2008 was approximately -0.25, this variable was categorized into < -0.25 or ≥ -0.25. Specific locality information other than ocean (North Pacific or North Atlantic) was unknown for 10 whales.

Supplementary Table 1. Anthropogenic stressors used to calculate cumulative impact scores. Stressors are those used in previous studies assessing human impacts on marine ecosystems[1,2].

Stressor
Demersal destructive fishing
Demersal nondestructive high bycatch fishing
Demersal nondestructive low bycatch fishing
Direct human impact (sum of the human coastal population)
Light pollution
Nutrient pollution
Oil rigs
Organic pollution
Pelagic high bycatch fishing
Pelagic low bycatch fishing
Sea surface temperature
Ultraviolet light reaching Earth's surface (climate change)

Supplementary Table 2. Mean difference in cumulative impact scores between 2013 and 2008 (2013 minus 2008) for 12 stressors based on the large marine ecosystems (LMEs) for each whale. The LMEs overlapping each whale's habitat were extracted from currently known distribution data for breeding and feeding grounds. If location of take or stranding was known, LMEs were selected based on the whale's most likely habitat. In cases where specific location of take or stranding is unknown beyond ocean, then impact scores from all possible LMEs used by the whale are included in the average.

ID	Species	Ocean	LMEs	Source(s)	SST Impact Score	Overall Mean Change in Impact Score
1001	Fin	Pacific	Sea of Okhotsk; Northern Bering-Chukchi Sea; West Bering Sea; East Bering Sea; Aleutians; Gulf of Alaska; California Current	4,5	-2.0182	-0.2703
1002	Fin	Pacific	Sea of Okhotsk; Northern Bering-Chukchi Sea; West Bering Sea; East	4,5	-2.0182	-0.2703

			Bering Sea; Aleutians; Gulf of Alaska; California Current			
1004	Fin	Atlantic (Netherlands)	Celtic-Biscay Shelf; North Sea; Iceland Shelf and Sea; Faroe Plateau; Norwegian Sea	4,6-9	0.0626	-0.1687
1005	Fin	Atlantic (Ireland)	Celtic-Biscay Shelf; North Sea; Iceland Shelf and Sea; Faroe Plateau; Norwegian Sea	4,6-9	0.0626	-0.1687
1006	Fin	Atlantic (Netherlands-Steinsham)	Celtic-Biscay Shelf; North Sea; Iceland Shelf and Sea; Faroe Plateau; Norwegian Sea	4,6-9	0.0626	-0.1687
1007	Fin	Atlantic (Netherlands-Steinsham)	Celtic-Biscay Shelf; North Sea; Iceland Shelf and Sea; Faroe Plateau; Norwegian Sea	4,6-9	0.0626	-0.1687
1008	Fin	Atlantic	Northeast US Continental Shelf; Scotian Shelf; Labrador-Newfoundland Shelf; Greenland Sea; Barents Sea; Norwegian Sea; Iceland Shelf and Sea; Faroe Plateau	4,6-9	0.2496	-0.0983
1009	Fin	Atlantic	Northeast US Continental Shelf; Scotian Shelf; Labrador-Newfoundland Shelf; Greenland Sea; Barents Sea; Norwegian Sea; Iceland Shelf and Sea; Faroe Plateau	4,6-9	0.2496	-0.0983
1010	Fin	Atlantic	Northeast US Continental Shelf; Scotian Shelf; Labrador-Newfoundland Shelf; Greenland Sea; Barents Sea; Norwegian Sea; Iceland Shelf and Sea; Faroe Plateau	4,6-9	0.2496	-0.0983
1011	Fin	Pacific (California)	California Current	10	-0.2685	-0.2549
1012	Fin	Pacific (Alaska)	Sea of Okhotsk; Northern Bering-Chukchi Sea; West Bering Sea; East Bering Sea; Aleutians; Gulf of Alaska; California Current	5,11-16	-2.0182	-0.2703
1013	Fin	Atlantic (Netherlands)	Northeast US Continental Shelf; Scotian Shelf; Labrador-Newfoundland Shelf; Greenland Sea; Barents Sea; Norwegian Sea; Iceland Shelf and Sea; Faroe Plateau	4,6-9	0.2496	-0.0983
1020	Humpback	Pacific (Alaska)	Insular Pacific-Hawaiian; East Bering Sea; West Bering Sea; Aleutians; Gulf of Alaska	17-22	-2.1866	-0.4475
1021	Humpback	Pacific (Alaska)	Insular Pacific-Hawaiian; East Bering Sea; West Bering Sea; Aleutians; Gulf of Alaska	17-22	-2.1866	-0.4475

1022	Humpback	Pacific (Alaska)	Insular Pacific-Hawaiian; East Bering Sea; West Bering Sea; Aleutians; Gulf of Alaska	17-22	-2.1866	-0.4475
1023	Humpback	Atlantic	Scotian Shelf; Labrador-Newfoundland Shelf; Canadian Eastern Arctic-West Greenland; Celtic-Biscay Shelf; Greenland Sea; Iceland Shelf and Sea; Norwegian Sea; Barents Sea; Caribbean Sea; Canary Current	23-28	0.9706	0.0067
1040	Blue	Atlantic	Scotian Shelf; Labrador-Newfoundland Shelf; Canadian Eastern Arctic-West Greenland; Iceland Shelf and Sea; Faroe Plateau; Norwegian Sea; Celtic-Biscay Shelf	29,30	0.5969	-0.0540
1041	Blue	Atlantic	Scotian Shelf; Labrador-Newfoundland Shelf; Canadian Eastern Arctic-West Greenland; Iceland Shelf and Sea; Faroe Plateau; Norwegian Sea; Celtic-Biscay Shelf	29-32	0.5969	-0.0540
1042	Blue	Atlantic	Scotian Shelf; Labrador-Newfoundland Shelf; Canadian Eastern Arctic-West Greenland; Iceland Shelf and Sea; Faroe Plateau; Norwegian Sea; Celtic-Biscay Shelf	29-32	0.5969	-0.0540
1043	Blue	Pacific (California)	California Current; Pacific Central-American Coastal	33-35	0.4326	0.2371

Supplementary Table 3. Evaluation of linear mixed-effects models of percent cortisol above baseline (baselevcort) in earwax of large baleen whales during 1900-1999. Reduced models were compared to the full model using likelihood ratio tests (LR; $\chi2$-squared distributed) and Akaike Information Criterion (AIC) after sequentially dropping covariates and assessing significance (P value < 0.05). Final model in bold; d.f. = degrees of freedom; sex/agecat3 = interaction term. Agecat3 = three age categories (calf/juvenile, subadult, adult); yearlywhalect = number of whales harvested each year; sstcont = yearly deviation from mean of sea surface temperatures over the years 1971-2000.

Model predictors for percent cortisol above baseline (baselevcort)	Model	Log-likelihood	AIC	LR	LR d.f.	P value
Sex+agecat3+sex/agecat3+yearlywhalect+sst	Full	-3580.2	7178.5	--	--	--
Sex+agecat3+sex/agecat3+sst	A	-3585.9	7187.8	11.36	1	0.001
**Sex+agecat3+sex/agecat3+yearlywhalect**	**B**	**-3580.9**	**7177.8**	**2.82**	**1**	**0.254**
Yearlywhalect	C	-3586.4	7180.7	12.28	5	0.031
SSTcont	D	-3599.1	7206.1	37.68	5	<0.001
Sex+agecat3+sex/agecat3	E	-3585.9	7185.8	11.36	2	0.003

Supplementary Table 4. Description of independent predictor variables used to initially evaluate cortisol levels in earwax plugs of large whales.

Variable name	Variable type
Species	Categorical
Fin	
Humpback	
Blue	
Sex	Categorical
Male	
Female	
Agecat3 (Three categories in years)	Categorical
<11 (Calf/Juvenile)	
11-24.9 (Subadult)	
≥25 (Adult)	
Sex X Age Interaction term	
Ocean	Categorical
Pacific	
Atlantic	
Proportion (%) harvested by ocean (Pacific or Atlantic)	Categorical
<10	
10-20	
>20	
Proportion (%) harvested 1900-1999 (based on mean of proportions from each decade)*	Categorical
<10	
≥10	
Number of whales harvested each year by species	Continuous
Sea surface temperature deviations from baseline mean of years 1971-2000	Continuous

* - Original data source did not differentiate between Pacific and Atlantic Oceans.

Supplementary Figure 1. Interaction of sex (male, solid line; female, dashed line) and age groups on levels of % baseline-corrected earplug cortisol in large baleen whales. Error bars are ± s.e.m.

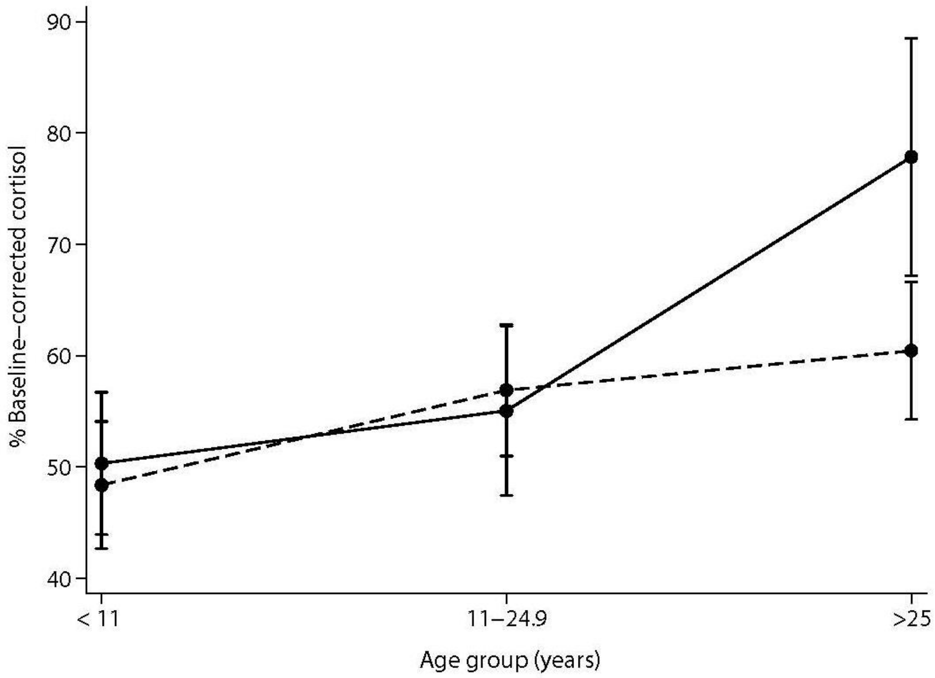

References

1 Halpern, B. S. *et al.* Spatial and temporal changes in cumulative human impacts on the world's ocean. *Nature Communications* **6**, doi:Artn 761510.1038/Ncomms8615 (2015).

2 Halpern, B. S. *et al.* A global map of human impact on marine ecosystems. *Science* **319**, 948-952, doi:10.1126/science.1149345 (2008).

3 Fisheries., N. O. a. A. A. *Humpback whale (Megaptera novaeangliae)*, <http://www.nmfs.noaa.gov/pr/species/mammals/whales/humpback-whale.html

4 Edwards, E. F., Hall, C., Moore, T. J., Sheredy, C. & Redfern, J. V. Global distribution of fin whales Balaenoptera physalus in the post-whaling era (1980-2012). *Mammal Rev* **45**, 197-214, doi:10.1111/mam.12048 (2015).

5 Mizroch, S. A., Rice, D. W., Zwiefelhofer, D., Waite, J. & Perryman, W. L. Distribution and movements of fin whales in the North Pacific Ocean. *Mammal Review* **39**, 193-227 (2009).
6 Seipt, I. E., Clapham, P. J., Mayo, C. A. & Hawvermale, M. P. Population Characteristics of Individually Identified Fin Whales Balaenoptera-Physalus in Massachusetts Bay. *Fish Bull* **88**, 271-278 (1990).
7 Skern-Mauritzen, M., Johannesen, E., Bjorge, A. & Oien, N. Baleen whale distributions and prey associations in the Barents Sea. *Mar Ecol Prog Ser* **426**, 289-301, doi:10.3354/meps09027 (2011).
8 Víkingsson, G. A. *et al.* Distribution and abundance of fin whales (Balaenoptera physalus) in the Northeast and Central Atlantic as inferred from the North Atlantic Sightings Surveys 1987–2001. *NAMMCO Sci. Publ* **7**, 49-72 (2009).
9 Berube, M. *et al.* Population genetic structure of North Atlantic, Mediterranean Sea and Sea of Cortez fin whales, Balaenoptera physalus (Linnaeus 1758): analysis of mitochondrial and nuclear loci. *Mol Ecol* **7**, 585-599, doi:DOI 10.1046/j.1365-294x.1998.00359.x (1998).
10 Falcone, E. A. & Schorr, G. S. Distribution and demographics of marine mammals in SOCAL through photoidentification, genetics, and satellite telemetry. (Monterey, California. Naval Postgraduate School, 2014).
11 Moore, S., Waite, J., Friday, N. & Honkalehto, T. Distribution and comparative estimates of cetacean abundance on the central and south-eastern Bering Sea shelf with observations on bathymetric and prey associations. *Progr Oceanogr* **55**, 249-262 (2002).
12 Moore, S. E., Stafford, K. M., Mellinger, D. K. & Hildebrand, J. A. Listening for large whales in the offshore waters of Alaska. *Bioscience* **56**, 49-55 (2006).
13 Sirovic, A., Williams, L. N., Kerosky, S. M., Wiggins, S. M. & Hildebrand, J. A. Temporal separation of two fin whale call types across the eastern North Pacific. *Mar Biol* **160**, 47-57, doi: 10.1007/s00227-012-2061-z (2013).
14 Soule, D. C. & Wilcock, W. S. D. Fin whale tracks recorded by a seismic network on the Juan de Fuca Ridge, Northeast Pacific Ocean. *J Acoust Soc Am* **133**, 1751-1761, doi:10.1121/1.4774275 (2013).
15 Stafforda, K. M., Mellinger, D. K., Moore, S. E. & Fox, C. G. Seasonal variability and detection range modeling of baleen whale calls in the Gulf of Alaska, 1999-2002. *J Acoust Soc Am* **122**, 3378-3390, doi:10.1121/1.2799905 (2007).
16 Zerbini, A. N., Waite, J. M., Laake, J. L. & Wade, P. R. Abundance, trends and distribution of baleen whales off Western Alaska and the central Aleutian Islands. *Deep-Sea Res Part I-Oceanogr Res Pap* **53**, 1772-1790, doi:10.1016/j.dsr.2006.08.009 (2006).
17 Baker, C. S. *et al.* Population structure of nuclear and mitochondrial DNA variation among humpback whales in the North Pacific. *Mol Ecol* **7**, 695-707, doi:DOI 10.1046/j.1365-294x.1998.00384.x (1998).
18 Barlow, J. *et al.* Humpback whale abundance in the North Pacific estimated by photographic capture-recapture with bias correction from simulation studies. *Mar Mamm Sci* **27**, 793-818, doi:10.1111/j.1748-7692.2010.00444.x (2011).
19 Calambokidis, J. *et al.* SPLASH: Structure of populations, levels of abundance and status of humpback whales in the North Pacific. *Unpublished report submitted by Cascadia Research Collective to USDOC, Seattle, WA under contract AB133F-03-RP-0078 [available from the author]* (2008).
20 Calambokidis, J. *et al.* Movements and population structure of humpback whales in the North Pacific. *Mar Mamm Sci* **17**, 769-794, doi:DOI 10.1111/j.1748-7692.2001.tb01298.x (2001).
21 Fleming, A. & Jackson, J. *GLOBAL REVIEW OF HUMPBACK WHALES (Megaptera novaeangliae)*. (2013).

22 Jackson, J. A. *et al.* Global diversity and oceanic divergence of humpback whales (Megaptera novaeangliae). *Proc Roy Soc B-Biol Sci* **281** (2014).

23 Palsbøll, P. *et al.* Stock structure and composition of the North Atlantic humpback whale, Megaptera novaeangliae. International Whaling Commission Scientific Committee, IWC, 135 Station Road, Impington, Cambridge, UK. (SC/53/NAH11, 2001).

24 Palsboll, P. J. *et al.* Genetic tagging of humpback whales. *Nature* **388**, 767-769, doi:Doi 10.1038/42005 (1997).

25 Palsboll, P. J. *et al.* Distribution of Mtdna Haplotypes in North-Atlantic Humpback Whales - the Influence of Behavior on Population-Structure. *Mar Ecol Prog Ser* **116**, 1-10, doi: 10.3354/Meps116001 (1995).

26 Reeves, R., Clapham, P. & Wetmore, S. American humpback whaling and humpback whale occurrence in the Cape Verde Islands, eastern Atlantic Ocean. *J Cet Res Manage* **4**, 235-253 (2002).

27 Stevick, P. T. *et al.* North Atlantic humpback whale abundance and rate of increase four decades after protection from whaling. *Mar Ecol Prog Ser* **258**, 263-273, doi: 10.3354/Meps258263 (2003).

28 Stevick, P. T. *et al.* Population spatial structuring on the feeding grounds in North Atlantic humpback whales (Megaptera novaeangliae). *J Zool* **270**, 244-255, doi:10.1111/j.1469-7998.2006.00128.x (2006).

29 Sears, R. & Calambokidis, J. *COSEWIC Assessment and Update Status Report on the Blue Whale, Balaenoptera Musculus, Atlantic Population, Pacific Population, in Canada*. (Committee on the Status of Endangered Wildlife in Canada, 2002).

30 Sears, R. & Larsen, F. Long range movements of a blue whale (Balaenoptera musculus) between the Gulf of St. Lawrence and West Greenland. *Mar Mamm Sci* **18**, 281-285, doi: 10.1111/j.1748-7692.2002.tb01034.x (2002).

31 Reeves, R. R., Smith, T. D., Josephson, E. A., Clapham, P. J. & Woolmer, G. Historical observations of humpback and blue whales in the North Atlantic Ocean: Clues to migratory routes and possibly additional feeding grounds. *Mar Mamm Sci* **20**, 774-786, doi: 10.1111/j.1748-7692.2004.tb01192.x (2004).

32 Sears, R. *et al. Photo identification of the Blue Whale (Balaenoptera musculus) in the Gulf of St. Lawrence, Canada*. Vol. 12 (1990).

33 Calambokidis, J. & Barlow, J. Abundance of blue and humpback whales in the eastern North Pacific estimated by capture-recapture and line-transect methods. *Mar Mamm Sci* **20**, 63-85, doi: I 10.1111/j.1748-7692.2004.tb01141.x (2004).

34 Mate, B. R., Lagerquist, B. A. & Calambokidis, J. Movements of North Pacific blue whales during the feeding season off southern California and their southern fall migration. *Mar Mamm Sci* **15**, 1246-1257, doi: 10.1111/j.1748-7692.1999.tb00888.x (1999).

35 Reilly, S. B. & Thayer, V. G. Blue Whale (Balaenoptera-Musculus) Distribution in the Eastern Tropical Pacific. *Mar Mamm Sci* **6**, 265-277, doi:10.1111/j.1748-7692.1990.tb00357.x (1990).
